# Preparing Health Care Workers for a Cholera Epidemic, Dominican Republic, 2010

**DOI:** 10.3201/eid1711.110703

**Published:** 2011-11

**Authors:** Consuelo Mendoza, Elissa Meites, Elizabeth Briere, Jacqueline Gernay, Oliver Morgan, Nelson Rodriguez Monegro, for the Hospital Preparedness Working Group

**Affiliations:** Author affiliations: Ministerio de Salud Pública y Asistencia Social, Santo Domingo, Dominican Republic (C. Mendoza, N. Rodriguez Monegro); Centers for Disease Control and Prevention, Atlanta, Georgia, USA (E. Meites, E. Briere, O. Morgan); Pan American Health Organization, Santo Domingo (J. Gernay)

**Keywords:** enteric infections, cholera, medical staff, hospital, epidemics, Dominican Republic, bacteria, health care workers

**To the Editor:** On October 21, 2010, a case of cholera was laboratory confirmed in Haiti, and within 2 months, ≈120,000 cases, 60,000 hospitalizations, and 2,500 deaths were reported (*1*). On November 16, the first laboratory-confirmed case of cholera was reported in the Dominican Republic, a country of 10 million persons sharing the island of Hispaniola with Haiti (*2*). Although the Dominican Republic has more developed sanitary infrastructure than does Haiti, spread of cholera into the country was inevitable (*3**,**4*).

Before 2010, most health care workers (HCWs) in the Dominican Republic lacked firsthand experience or training in managing cholera patients (*5**,**6*). To improve response capacity, the Ministry of Health, in collaboration with international experts, rapidly produced clinical protocols for cholera diagnosis, treatment, and infection control (*7**,**8*). Pocket guides and posters were distributed throughout each region during October and November.

During December 1–17, field teams of public health physicians visited 67 critical resource hospitals (all 11 regional referral hospitals, all 20 large provincial hospitals, and 36 additional municipal hospitals across the country). Field teams met with hospital directors and staff, conducted a survey to assess recent cholera training and knowledge based on national protocols, and offered additional training materials and resources (*7*).

At each hospital, a convenience sample of all available HCWs (e.g., physicians, nurses, cleaning staff) was assembled to discuss cholera. After giving verbal consent, participants anonymously self-administered a standardized 20-question multiple-choice survey. Immediately afterward, answer keys and explanations were distributed for participants to review, along with supplemental training materials including videos, posters, and pocket guides.

Responses to questionnaires were scanned, scored, and analyzed by using Remark Office software version 8.0 (OMR Data Center, Gravic Inc., Malvern, PA, USA). The Centers for Disease Control and Prevention determined that this evaluation was not research because it was conducted as a public health response to an emergency.

Of 233 respondents who completed surveys, 125 (54%) were physicians, 33 (14%) licensed nurses, 57 (24%) auxiliary nurses, and 18 (8%) another type of hospital staff. At least 58 (25%) believed that at least 1 of their 100 most recent patients had illness that met the definition for a suspected case of cholera.

Eighty (34%) respondents answered >80% of questions correctly; 97 (42%) answered 60%–79% of questions correctly, and 56 (24%) answered <60% of questions correctly. Nationally, the average overall test score was 71%.

Most (174 [75%]) respondents reported having received cholera protocol training after the start of the outbreak in Haiti, and 146 (63%) already carried their personal copy of the national pocket guide to cholera diagnosis and treatment. Respondents who had received cholera training answered a mean of 76% of questions correctly, whereas respondents who had not received cholera training answered a mean of 62% of questions correctly. Of respondents who reported receiving training, the largest group was physicians (111 [64%]). Most (>90%) participants correctly answered questions about case definitions for suspected cholera and the need for immediate rehydration for all cholera patients (Table).

**Table:** Percentage of 233 hospital staff correctly answering cholera knowledge assessment questions near the start of a cholera epidemic in the Dominican Republic, 2010

Topic	No. (%)
Importance of rehydration	207 (89)
Suspected cholera case definitions	206 (88)
Nutrition for cholera patients	198 (85)
Ideal type of intravenous fluid	189 (81)
Infection control measures	175 (75)
Environmental cleaning	168 (72)
Risk factors for disease	157 (67)
Handling cadavers	146 (63)
Quantity and timing of intravenous fluid	128 (55)
Uses of bleach solution	128 (55)
Treatment of mild dehydration	125 (54)
Identification of severe dehydration	122 (52)
Treatment of severe dehydration	108 (46)
Disinfection methods	100 (43)

Most HCWs had received cholera protocol training through public health efforts, which improved knowledge in key topic areas. Improving health care response capacity is an important way to reduce cholera case-fatality rates (*9*). The World Health Organization recommends that intervention strategies ideally should aim to reduce case-fatality rates to <1% by ensuring access to treatment and controlling the spread of disease (*10*). As of December 18, 2010, cholera had been confirmed in 59 persons in the Dominican Republic; 46 of these persons were hospitalized, and none died (*1*). Efforts to prepare HCWs likely contributed to the initially low case-fatality rate by improving clinical case management and response capacity. These successful collaborative efforts to train HCWs strengthened relationships and communication within local, national, and international public health networks.

This analysis has at least 4 limitations. First, although all major hospitals in the country were visited and all geographic regions are represented, hospital staff were not randomly selected; thus, results are not generalizable to hospital staff in the country. Second, denominator information for types of HCWs was not available. Third, biases are possible in self-reported survey results. Finally, primary care centers, which may be important sites commonly visited by persons seeking acute medical care especially in rural areas, were not systematically assessed.

To strengthen the capacity of HCWs to respond to threats such as epidemic cholera, our approach included site visits, dissemination of national clinical guidelines, and rapid assessment of knowledge deficiencies that could be addressed by providing immediate training and educational materials. Training should involve not solely physicians but also hospital staff who may provide direct care to patients, such as nurses, and other personnel who may share infection control responsibilities. Hospital staff should maintain ongoing communication with public health leadership.

## References

[1] Centers for Disease Control and Prevention. Update on cholera—Haiti, Dominican Republic, and Florida, 2010. MMWR Morb Mortal Wkly Rep. 2010;59:1637–41. 21178947

[2] Ministry of Public Health of the Dominican Republic. Cholera—special bulletin number 10, December 1, 2010 [in Spanish] [cited 2011 Jul 31]. http://www.sespasdigepi.gob.do/documentos/Octubre%202010%20Colera%20Haiti/PDF/Boletines/Boletin_Colera_No%2010_2010_12_1.pdf

[3] Walton DA, Ivers LC. Responding to cholera in post-earthquake Haiti. N Engl J Med. 2011;364:3–5. 10.1056/NEJMp1012997 21142690

[4] Ali A, Chen Y, Johnson JA, Redden E, Mayette Y, Rashid MH, Recent clonal origin of cholera in Haiti. Emerg Infect Dis. 2011;17:699–701. 21470464 10.3201/eid1704.101973 PMC3377427

[5] Chin CS, Sorenson J, Harris JB, Robins WP, Charles RC, Jean-Charles RR, The origin of the Haitian cholera outbreak strain. N Engl J Med. 2011;364:33–42. 10.1056/NEJMoa1012928 21142692 PMC3030187

[6] Sack DA, Sack RB, Nair GB, Siddique AK. Cholera. Lancet. 2004;363:223–33. 10.1016/S0140-6736(03)15328-7 14738797

[7] Ministry of Public Health of the Dominican Republic. Clinical guide to the diagnosis and treatment of cholera [in Spanish] [cited 2011 Jul 31]. http://www.sespasdigepi.gob.do/documentos/Octubre%202010%20Colera%20Haiti/PDF/Guia%20de%20Diagnostico%20y%20Tratamiento%20Colera.pdf

[8] World Health Organization Global Task Force on Cholera Control. Cholera outbreak: assessing the outbreak response and improving preparedness. Geneva: The Organization; 2004.

[9] Ivers LC, Farmer P, Almazor CP, Leandre F. Five complementary interventions to slow cholera: Haiti. Lancet. 2010;376:2048–51. 10.1016/S0140-6736(10)62243-X 21146206

[10] World Health Organization Global Task Force on Cholera Control. Prevention and control of cholera outbreaks: WHO policy and recommendations. 2011 [cited 2011 Jul 31]. http://www.who.int/cholera/technical/prevention/control/en/index.html

